# Supplementary material for: Deviation of Latitude Cut: A Simple Sign to Differentiate Total Hip Arthroplasty from Hemiarthroplasty in Radiography
Source: J Clin Med. 2023 Sep 26;12(19):6218. doi: 10.3390/jcm12196218 (PMC10573713; doi:10.3390/jcm12196218)
Supplement: Supplementary file 1 [file jcm-12-06218-s001.zip › jcm-2600307-supplementary.pdf]

Table S1. Implants used for hip arthroplasty

Component		Product Name	Number
Total hip arthroplasty	Acetabular cup	Continuum (Zimmer Biomet, Warsaw, Indiana, USA)	13
		EP-FIT plus (Smith & Nephew, Andover, Massachusetts, USA),	3
		G7 (Zimmer Biomet)	2
		Trilogy (Zimmer Biomet, Warsaw, Indiana, USA)	1
		Plasmacup (Aesculap, Tuttlingen, Germany)	1
	Stem	Alloclassic (Zimmer Biomet)	7
		M/L Taper (Zimmer Biomet)	6
		SL-PLUS (Smith & Nephew)	3
		ARCOS (Zimmer Biomet)	2
		Bicontact (Aesculap)	1
		TaperLoc Microplasty (Zimmer Biomet)	1
Bipolar hemiarthroplasty	Bipolar cup	Bencox Bipolar Cup (Corentec, Cheoan, Korea)	8
		Multipolar Cup (Zimmer Biomet)	4
		RINGLOC Cup (Zimmer Biomet)	4
		Bipolar Prosthesis (Smith & Nephew)	3
		Bipolar cup (Lima, Udine, Italy)	1
	Stem	Bencox M stem (Corentec)	3
		Taperloc (Zimmer Biomet)	3
		Bencox II (Corentec)	2
		MIA stem (Smith & Nephew)	2
		Alloclassic (Zimmer Biomet)	2
		MUTARS (Implantcast Ltd, Buxtehude, Germany)	1
		Taperloc Microplasty (Zimmer Biomet)	1
		Benfix Long (Corentec)	1
		C2 (Lima)	1
		Bencox ID stem (Corentec)	1
		CLS (Zimmer Biomet)	1
		M/L Taper (Zimmer Biomet)	1
		SL-plus (Smith & Nephew)	1
